# Control of zoonotic cutaneous leishmaniasis vector, *Phlebotomus papatasi*, using attractive toxic sugar baits (ATSB)

**DOI:** 10.1371/journal.pone.0173558

**Published:** 2017-04-20

**Authors:** Abedin Saghafipour, Hassan Vatandoost, Ali Reza Zahraei-Ramazani, Mohammad Reza Yaghoobi-Ershadi, Yavar Rassi, Moharram Karami Jooshin, Mohammad Reza Shirzadi, Amir Ahmad Akhavan

**Affiliations:** 1 Department of Medical Entomology and Vector Control, School of Public Health, International Campus (IC -TUMS), Tehran University of Medical Sciences, Tehran, Iran; 2 Department of Medical Entomology and Vector Control, School of Public Health, Tehran University of Medical Sciences, Tehran, Iran; 3 Qom Provincial Health Center, Qom University of Medical Sciences, Qom, Iran; 4 Communicable Diseases Management Center, Ministry of Health and Medical Education, Tehran, Iran; Instituto Oswaldo Cruz, BRAZIL

## Abstract

**Introduction:**

Attractive Toxic Sugar Baits (ATSB) is a new vector control method that meets Integrated Vector Management (IVM) goals. In an experimental design, this study aimed to determine effects of ATSB on control of *Phlebotomus papatasi*, as a main vector of Zoonotic Cutaneous Leishmaniasis (ZCL), in Qom Province, center of Iran.

**Methods:**

In a cross-sectional design, boric acid was mixed with brown sugar solution and tested as toxic baits for *P*. *papatasi*. Two methods were utilized to use the baits: (a) spraying ATSB on vegetation, bushes, and shrubs; and (b) setting ATSB-treated barrier fences in front of colonies at 500 m distance from the houses in outskirts of villages. In order to examine the residual efficacy rate of ATSB-treated barrier fences, the bioassay test was used. Density of *P*. *papatasi* sandflies was measured using sticky and light traps biweekly. For data analysis, Mann-Whitney U Test and Kruskal-Wallis were used. Results ATSB-treated barrier fences led to 3 times reduction in *P*. *papatasi* population. Besides that, ATSB spraying on plants led to more than 5 times reduction in *P*. *papatasi* population.

**Conclusions:**

Comparing the incidence of leishmaniasis in treated villages before and after the study showed that the incidence was statistically reduced. Therefore, ATSB is an effective method to control vectors and prevent leishmaniasis.

## Introduction

Leishmaniasis is caused by a protozoa parasite belonging to (over 20) *Leishmania* species that is transmitted to humans by infected female phlebotomine sand fly bites (Center for Disease Control and Prevention) [1].Cutaneous leishmaniasis (CL) is one of the major vector-borne diseases in Iran [2].The *P*. *papatasi* (Diptera: Psychodidiae) is the main and proven vector, and *Leishmania (Leishmania) major* is the causative agent of Zoonotic Cutaneous Leishmaniasis (ZCL) in Iran [3].Currently, there are different methods to control sand flies. The most important measure used to control adult sand flies (Diptera: Psychodidae: Phlebotominae) is the use of insecticides (mostly Pyrethroids) in different modes. Insecticides are used as sprays in residual dwellings and animal shelters, space-spraying, insecticide-treated nets, impregnated dog collars, and personal protection through application of repellents/insecticides. As breeding-sites of sand flies are generally unknown, control measures that act specifically against immature groups are not feasible; although the effectiveness of some biological and chemical agents has been demonstrated in laboratory evaluations in this regard [4]. One of the new tools for vector control program that has led to significant reduction in vectors populations is the use of attractive toxic sugar baits (ATSB) [5–7]. This method has been used successfully to control mosquitoes and sand flies in many regions such as Africa (African-Syrian Rift Valley), the Middle East (Jordan valley, Israel) and America [7–9]. The utilization of ATSB to control mosquitoes in initial field trials resulted in significant reductions in sand fly populations [8,10]. The field evaluations have effectively controlled sand flies through ATSB application to patches of plants [11] and as barrier fences in areas lacking vegetation for spraying [8]. The latter method has been remarkably successful because both male and female sand flies, like other biting flies, depend on sugar from plants and sometimes honeydews for survival [12–14]. ATSB, commonly, consist of oral pesticides such as boric acid, fruit juice, brown sugar, and water [7]. In different studies, these baits have been used in certain ways, including ATSB spraying on plant bushes, barrier fences soaked with ATSB solution, and bait stations [8].Two of these methods were tested in this study: (a) barrier fences soaked with ATSB set in front of rodent colonies, (b) ATSB spraying on patches of vegetation, and(c) bait stations. The effectiveness of these methods was compared in terms of the *P*. *papatasi* populations reduction in an endemic area in Central Iran, Qom province. The type of cutaneous leishmaniasis is ZCL in this area of Iran [15].

## Materials and methods

### Study area

The study was performed from April to November 2015 in rural areas of the Markazi District (34o09'–35o11' N latitude and 50o06'–51o58' E longitude, 1500 m above the sea level) of the Qom Province, 125 km off the south of Tehran. In these areas, immigration rate is very low, the indigenous people have access to tap water, and ZCL and communicable diseases such as tuberculosis [16], whooping cough and meningitis [17] are prevalent. Rodent burrows can be easily found in the very vicinity of villages in this district, where *P*. *papatasi* as the main vector and *Meriones libycus* as a reservoir of ZCL abound [15, 18]. Other rodents such as *Nesokia indica*, *Allactaga elater* and *Mus musculus* are also common inhabitants and majority of them are constantly exposed to *L*. *major* [18]. Besides these rodents, foxes and rabbits are also frequently seen. Economy of the district is primarily dependent on agriculture (e.g. wheat, corn and barley) and livestock (cattle, goats, sheep, camels, and horses). The district soil is chiefly made of clay, and tamarix shrubs (T. aphylla) represent the predominant vegetation. Other plant species that grow in this district are *Astragalus spp* and *Crataegus* hawthorn. The climate is arid, with an annual rainfall of 150 mm. The average monthly maximum and minimum relative humidity are 84% and 28% in December and June, respectively. The average annual minimum and maximum temperature are -16.5°c and 49°c in January and July, respectively.

### Monitoring sand flies

Density of *P*. *papatasi* in treatment and control villages was measured once every 15 days using sticky paper traps and light traps from April to November 2015. On monitoring days, sand flies were caught overnight at each village with 2 CDC-like miniature light traps and 60 sticky traps. Traps were hung, approximately 30 cm above than the ground, on the indoor walls of stables and walls of the homes near the treatment area. Sand flies in trap nets were kept at 0–5°C for one to two hours while being transported to the laboratory. The sand flies caught were preserved in 70% alcohol and then mounted in a drop of *Puri medium* [19]. Species identification was carried out using the keys of Lewis (1982), Nadim and Javadian (1976), and Seyedi-Rashti and Nadim (1992). Experiments were conducted from early April to the end of November, and the sites were monitored once every 15 days. In addition, we compared the population of sand flies with the statistics of the previous year. Sand fly collection in the previous year was carried out by the same team and in the same manner and we have observed that the villages were exposed to sand fly communities were similar abundance before treatments. In addition to comparing sand fly populations before and after the study in treatment villages, for each treatment village, a control village was selected.

### Preparation of ATSB solutions

Bait solution was prepared as described by Müller et al. [20]. For the treatment sites, it consisted of 10% w/v brown sugar, 1.0% w/v boric acid [20], and water (i.e. ATSB). In control sites, we used food dye (instead of boric acid), 10% w/v brown sugar and water (i.e. attractive sugar baits without toxin or ASB).

### Presentation of ATSB at different sites

Five villages were selected in the study area. For every two villages in which ATSB was presented, an adjacent village was chosen to serve as an untreated control site. Kooh-Sefid and Faraj-Abad Villages were chosen for ATSB and ASB treated barrier fences method, and Jafar-Abad and Said-Abad villages were selected for ATSB and ASB sprayed on vegetation. Ali-Abad village was considered as an untreated site. These villages were chosen for their similarities in the number of sand flies, incidence of ZCL in previous years, climate conditions (such as temperatures and humidity), topographical features of the area, geographical conditions, the approximate number of rodents’ burrows near the villages, the distance of the rodents’ burrows from the village (i.e. 500 meters away from the last house of the village), the human population living in the villages, and the vegetation type and cover. Solution with toxin was used for the experimental villages and without toxin for the control villages.

### ATBS-treated barrier fences

In the two villages of Kooh-Sefid and Faraj-Abad, rolls of semi-rigid plastic nets (with 100 cm width and 1x2 mm thickness) were cut, depending on largeness of rodent colonies, and used for building a bait barrier fence (mesh size 156 hole/inch, hole/cm 2 = 25, denier = 75). Strips of cotton cloth (5x60 cm^2^) were connected transversally to the net and their ends were folded around the margins and stapled. The strips were then fixed to the net at intervals of 20 cm. After fixing the cloth strips, the net was rolled into a cylinder that was dipped into a bucket containing ATSB solution or ASB. In this method, which was conducted in the Kooh-Sefid and Faraj-Abad villages, the number, location, and extent of rodent colonies around the villages were identified and characterized. Then, net fences treated with ATSB were installed in the close front of their colonies at a distance of 500 m from the houses in the outskirt of the village. In the treatment and control sites, barrier fences were set up on the ground in front of the rodent colonies, using about 150 cm metal rods that were hammered 50 cm deep into the ground in suitable distances. This prevented from the movement of sand flies towards the village. In order to test the residual efficacy rate of ATSB-treated barrier fences, the bioassay test was used.

### Bioassay test

**Fig 1:**
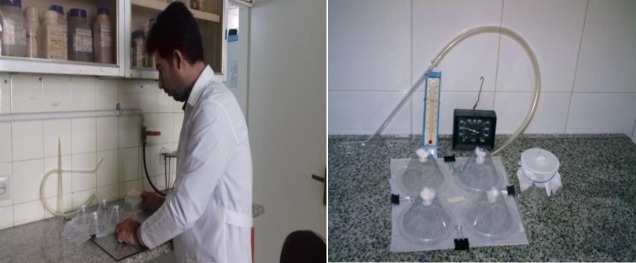
Bioassay test to determine residual efficacy rate of ATSB-treated barrier fence in Markazi district, Qom province, Iran, 2015.

The ATSB-treated barrier fence nets (25 cm × 25 cm) were removed from the installed barriers in the villages and were fixed by fiberglass sheets (with a dimension of 25 × 25 cm and four quarters of a circle with equal intervals on the nets to stable the cones). The diameter of each circle was 9 cm, the distance from one circle to another 3 cm, and the distance from the circles to the edge 2 cm. Both the fiberglass sheets were connected and immobilized from one side with scotch tape. In order to enhance and improve test accuracy and prevent from pollution by various chemicals frameworks, disposable plastic sheets on the internal surfaces were installed with the same dimensions and the size of the frame and the disposable plastic sheets were fixed to the internal surfaces of frameworks by using scotch tape. The removed ATSB-treated nets (25 × 25 cm) were placed into the plastic sheets. The cones placed on the quarters of the circle of nets and the other fiberglass frame was fixed with clamps (Fig 1). Sand flies were immediately transferred to the laboratory, and after 1 h, the sensitivity test was completed. To determine the residual effect of ATSB bait on barrier fences, bioassay test (Cone test) was used in treatment village. World Health Organization protocol, using conical chamber at biweekly interval, was used for that end [21]. Hence, in the case of reduced residual effect of ATSB on barrier fences, they were impregnated with ATSB solution again. In each conical tube, 10 sand flies were gently released into any cone at three replicates. Overall, 120 sandflies were tested each time by using an aspirator with a minimum exposure of 3 min on the treated surfaces. Three replicates of untreated surfaces were used as negative controls (ASB barrier fence nets) simultaneously. After 3-minute exposure, the sand flies were transferred into clean cups and kept in suitable conditions (25±2°C and 80%±10% relative humidity) by placing a wet towel over the cups containing the sand flies for 24 hours. After the exposure time, both living and dead sand flies were transferred in netted cups to the laboratory and their mortality was recorded after 24 h. If mortality rate in control tests will be between 5 to 20%, then the mortality rates were corrected using Abbott’s formula. One-way ANOVA test was used to compare the residual activities in sprayed surfaces. The criterion for residual effect of tested insecticide was based on the mortality rate; if this rate had decreased to 60–65% [21], the bioassay was stopped and the data analyzed. To control for confounding effect of sandflies deaths due to catching process, sandflies that were released into each cone were caught again gently and carefully by an aspirator. The obtained results were recorded according to the survival and mortality rate of sand flies. These tests were carried out from 5 days after spraying and repeated every 15 days until the mortality rate had decreased to 60–65% [21]. In addition, the barrier fences were reviewed and repaired every 15 days. Bioassay test was conducted on wild sand flies that were collected in one village near the study area.

### ATSB sprayed on vegetation

In the second pair of villages, Jafar-Abad as an experimental village and Said-Abad as a control village, ATSB solution was sprayed, using a HODSON X-Pert®110, on patches of vegetation around the villages 500 meters off the last house and near to the rodents’ burrows. The dominant plant species in these areas was *Tamarisk aphylla* [22], which were completely sprayed within the 500 m from the last houses surrounding the village. Müller and Schlein showed that flowering *Tamarix nilotica* is a highly attractive sugar source for sand flies [14]. Based on an attraction index, the top three attractive plants in this study were flowering *Ochradenus baccatus*, *Prosopis farcta*, and *Tamarix nilotica* [8]. The spraying was performed around these two villages (Jafar-Abad & Said-Abad) from May to November 2015. In this pair of sites, ATSB solution was sprayed on patches of the vegetation, bushes, and shrubs around the village, using a HODSON X-Pert®110, until 500 m from the last houses so that all the trees were wet. The spraying of the vegetation, bushes, and shrubs around the villages was repeated once every 30 days. Every 15 days, the sand fly population was determined using two light traps and 60 sticky paper traps (bedroom, bathroom, toilets, hall, stables, outdoor = 30 sticky traps on the exterior walls of the last houses). Solution with toxin was used for the experimental village and without toxin for the control village.

### Data on human infection

To determine the incidence of the disease in 2015, the following villages were selected: Kooh-Sefid and Faraj-Abad for ATSB and ASB-treated barrier fences, Jafar-Abad and Said-Abad for ATSB and ASB sprayed on vegetation and Ali-Abad with no intervention. Active screenings were seasonally conducted by visiting all residents in the villages to find active wounds of ZCL. To understand the epidemiology of the disease, a questionnaire was completed by patients that asked for their age, gender, history of travel at the time of transmission, number of lesions, sites of lesions, and seasons of the disease prevalence. Interestingly, there were inhabitants with no traveling history to other endemic areas but with ulcers. Previous research using ITS1-PCR method showed that *Leishmania major* was the causative agent for these patients’ ulcers [15, 18].

### Ethics statement

The study was approved by and carried out under the guidelines of Ethical Committee of School of Public Health, Tehran University of Medical Sciences, Tehran, Iran. The study was carried out in a preserved desert land and required permissions were obtained from Ministry of Environment. However, the field in which study was conducted had no endangered or protected species.

### Statistical analysis

Histogram plots were used to check for normality of data. (If the data is normally distributed, the curve will be a bell–shaped curve and ANOVA can be used for analysis. In addition, Shapiro-Wilk and Kolmogorov- Simonov tests showed that the distribution was normal (p = 0.01). In an abnormal distribution, the histogram plot will not be bell-shaped. The data was abnormally distributed in the present study. Kruskal-Wallis H Test is considered the nonparametric alternative for one-way ANOVA, and an extension of the Mann-Whitney U Test allows comparison of more than two independent groups. So, Kruskal-Wallis H Test was used one site with others. The Mann-Whitney U Test was utilized to compare the total frequencies of *P*. *papatasi* sand flies that were collected every 15 days using sticky paper traps and light traps from April to November 2015 in rural areas (treatment and control villages). Also, for comparing the effectiveness of this method in the five villages (two treatment and two control villages and one untreated village), the Kruskal-Wallis Test was used. Then, in order to determine the effect of the method on the disease, the incidence of the disease before 2014 and after 2015 was examined. Statistical analyses were performed using the software SPSS 16. P-values less than 0.05 were considered as significant.

## Results

Firstly, the species composition of sand flies was determined. In this study, 10 species were collected and identified: *P*. *papatasi*, *P*. *segenti*, *P*. *caucasicus*, *P*. *alexandri*, *P*. *caucasicus* group, *Sergentomyia sintoni*, *S*. *dentata*, *S*. *clydei*, *S*. *theodori*, *S*. *pawlowski*. The species composition was similar in the five villages before and after treatment (p < 0.05) (Table 1).

**Table 1:** The density of *Phlebotomus papatasi* collected in our area study before treatment.

Density	Male	Female	Total
**Sites**	**No. (%)**	**No. (%)**	**No. (%)**
Koohsefid	1175 (66.31)	597 (33.69)	1772 (21.27)
Faraj abad	864 (51.22)	823 (48.78)	1687 (20.25)
Jafar abad	601 (54.79)	496 (45.21)	1097 (13.17)
Said abad	983 (52.82)	878 (47.18)	1861 (22.35)
Ali abad	1058 (55.33)	854 (44.67)	1912 (22.96)

In the case of ATSB sprayed on the vegetation sites and in post-treatment analysis, there was a significant fall (3.45 times) in the ratio of *P*. *papatasi* populations compared to the pre-treatment numbers in the previous year (Table 2). In the treatment village, there were -73.97% (3.84 times), -68.87% (3.21 times), and -71.01% (3.45 times) reductions in the initial densities of *P*. *papatasi* indoors, outdoors, and in total, respectively (Table 2).

**Table 2:** The comparison of *P*. *papatasi* frequency with sticky traps before and after treatment in Jafar-Abad, Markazi district, Qom province, 2014–2015.

Site	Indoor			Outdoor			Total		
Time and type of Intervention	Male	Female	Total	Male	Female	Total	Male	Female	Total
	No. (%)	No. (%)	No. (%)	No. (%)	No. (%)	No. (%)	No. (%)	No. (%)	No. (%)
Before spraying ATSB	167 (23.36)	294 (63.77)	461 (42.02)	434(68.24)	202 (31.76)	636 (57.98)	601 (54.79)	496 (45.21)	1097 (100)
After spraying ATSB	31 (25.83)	89 (74.17)	120 (37.74)	152 (76.77)	46 (23.23)	198 (62.26)	183 (57.55)	135 (42.45)	318 (100)
Percentage change	-81.44	-69.73	-73.97	-64.98	-77.23	-68.87	-69.55	-72.78	-71.01
Ratio change	5.39	3.30	3.84	2.86	4.39	3.21	3.28	3.67	3.45

Based on the results in according to Table 3, the frequency of *P*. *papatasi* collected by indoors and outdoors sticky traps in Jafar-Abad (plants, bushes, and shrubs sprayed with ATSB), showed a decline in comparison to Said-Abad (plants, bushes, and shrubs sprayed with ASB). In fact, in Jafar-Abad, the percentage change in sand flies population reached to -77.78%, -81.51%, and -80.26% indoors, outdoors, and in total, respectively (Table 3). In addition, the frequency of *P*. *papatasi* collected by light traps considerably reduced (Table 4). According to statistical tests, in the site with ATSB sprayed on vegetation, compared with the ASB sprayed site, not only the ratio change of *P*. *papatasi* density reduced more than 5 times (Tables 3 and 4), this reduction was also statistically significant (p = 0.029).

**Table 3:** The comparison of *P*. *papatasi* frequency indoors and outdoors in Jafar-Abad and Said-Abad using sticky traps (plants, bushes, and shrubs sprayed with ATSB and ASB, Markazi district, Qom province, 2015.

Site	Indoor			Outdoor			Total		
Site and type of Intervention	Male	Female	Total	Male	Female	Total	Male	Female	Total
	No. (%)	No. (%)	No. (%)	No. (%)	No. (%)	No. (%)	No. (%)	No. (%)	No. (%)
Said-Abad (ASB)	194 (35.93)	346 (64.07)	540 (33.52)	658 (61.44)	413 (38.56)	1071 (66.48)	852 (52.89)	759 (47.11)	1611 (100)
Jafar-Abad (ATSB)	31 (25.83)	89 (74.17)	120 (37.74)	152 (76.77)	46 (23.23)	198 (62.26)	183 (57.55)	135 (42.45)	318 (100)
Percentage change	-84.02	-74.28	-77.78	-76.90	-88.86	-81.51	-78.52	-82.21	-80.26
Ratio change	6.25	3.89	4.50	4.33	8.98	5.41	4.65	5.62	5.07

**Table 4:** The comparison of *P*. *papatasi* frequency indoors and outdoors in Jafar-Abad and Said-Abad using light traps (plants, bushes, and shrubs sprayed with ATSB and ASB, Markazi District, Qom province, 2015.

Site	Indoor			Outdoor			Total		
Site and type of Intervention	Male	Female	Total	Male	Female	Total	Male	Female	Total
	No. (%)	No. (%)	No. (%)	No. (%)	No. (%)	No. (%)	No. (%)	No. (%)	No. (%)
Said-Abad (ASB)	63 (26.92)	171 (73.08)	234 (29.43)	389 (69.34)	172 (30.66)	561 (70.57)	452 (56.85)	343 (43.15)	795 (100)
Jafar-Abad (ATSB)	35 (72.92)	13 (27.08)	48 (32.21)	58 (57.43)	43 (42.57)	101 (67.79)	93 (62.42)	56 (37.58)	149 (100)
Percentage change	-44.45	-92.40	-79.49	-85.09	-75	-82	-79.42	-83.67	-81.26
Ratio change	1.80	13.15	4.87	6.71	4	5.55	4.86	6.12	5.34

**Fig 2:**
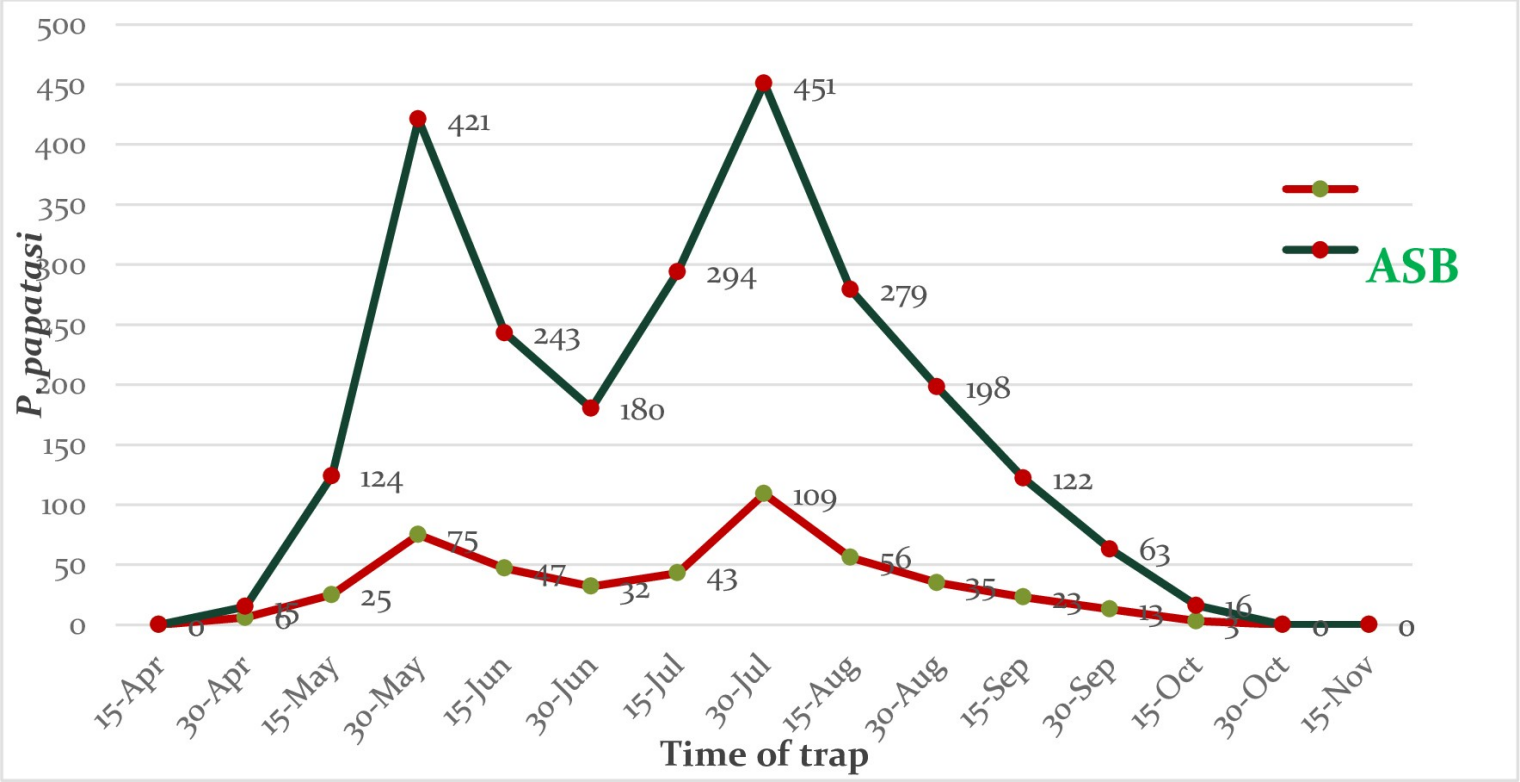
Number of *P*. *papatasi* sand flies collected from sprayed vegetation in the Markazi district, Qom province, 2015, Iran.

**Fig 3:**
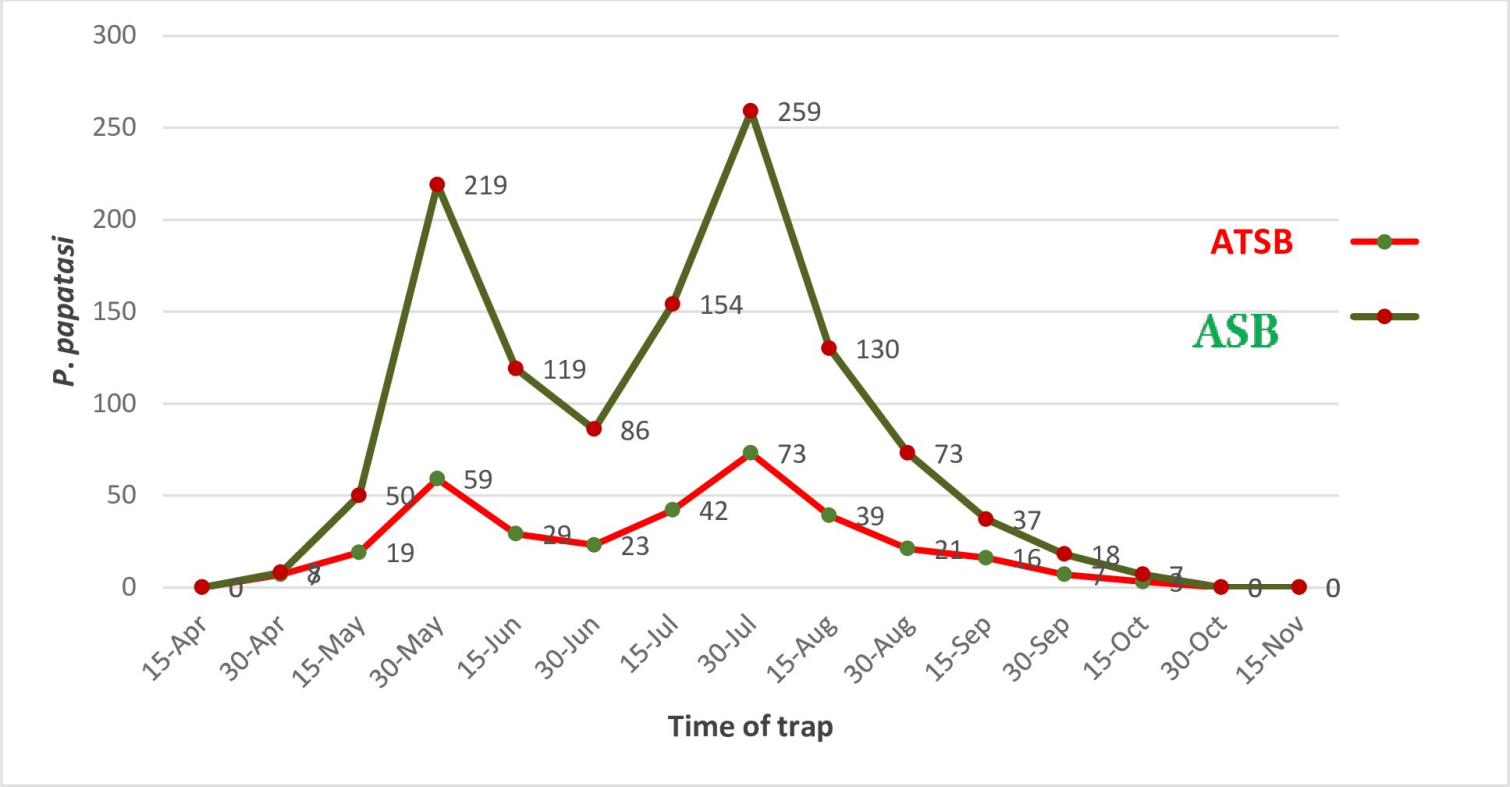
Number of P. papatasi sand fly collected from treated barrier fences in Markazi District, Qom province, 2015, Iran. -Exposed to ATSB, -exposed to ASB.

Also, the graphs of monthly activity of *P*. *papatasi* collected from treated barrier fences and treated vegetation show the reduction of their density in the sites where sand flies were exposed to ATSB (Fig 2 and Fig 3). In the treatment village, after the setup of the ATBS-treated barrier fences, the Ratio changes Percentage changes and of reduction in the initial densities of *P*. *papatasi* was 8.79 (-88.68%), 7.85 (-87.27%), and 8.28 (-87.93%) times indoors, outdoors and in total (Table 5). Table 1 demonstrates the percentage change in the *P*. *papatasi* population in the village following the barrier fences. Compared to pre-treatment numbers of the previous year, there was a considerable reduction in the *P*. *papatasi* density in post–ATSB application as barrier fences in treatment site (-87.93%) (Table 5).

**Table 5:** The comparison of *P*. *papatasi* frequency caught with sticky traps before and after treatment (ATSB treated barrier fences) in Kooh-Sefid, Markazi District, Qom province, 2014–2015.

Site	Indoor			Outdoor			Total		
Time and type of Intervention	Male	Female	Total	Male	Female	Total	Male	Female	Total
	No. (%)	No. (%)	No. (%)	No. (%)	No. (%)	No. (%)	No. (%)	No. (%)	No. (%)
Before ATSB treated barrier fences	559 (64.92)	302 (35.08)	861 (48.59)	616 (67.62)	295 (32.38)	911 (51.41)	1175 (66.31)	597 (33.69)	1772 (100)
After ATSB treated barrier fences	62 (63.27)	36 (36.73)	98 (45.79)	85 (73.28)	31 (26.72)	116 (54.21)	147 (68.69)	67 (31.31)	214 (100)
Percentage change	-88.91	-88.08	-88.68	-86.20	-89.49	-87.27	-87.49	-88.78	-87.93
Ratio change	9.02	8.39	8.79	7.25	9.52	7.85	8	8.91	8.28

The comparison of *P*. *papatasi* frequency indoors and outdoors in Kooh-Sefid and Faraj-Abad (ATSB and ASB-treated barrier fences) using sticky traps showed that there was a considerable reduction in *P*. *papatasi* density with ATSB-treated barrier fences in the treatment site, compared to the control site where ASB were used (-69.94%). The Ratio change showed that the population of *P*. *papatasi* reduced more than 3 times (3.33 times) (Table 6).

**Table 6:** The comparison of *P*. *papatasi* frequency indoors and outdoors of Kooh-Sefid and Faraj-Abad using sticky traps (ATSB and ASB-treated barrier fences), Markazi district, Qom province, 2015.

Site	Indoor			Outdoor			Total		
Site and type of Intervention	Male	Female	Total	Male	Female	Total	Male	Female	Total
	No. (%)	No. (%)	No. (%)	No. (%)	No. (%)	No. (%)	No. (%)	No. (%)	No. (%)
Faraj-Abad (ASB treated barrier fences)	118 (34.10)	228 (65.90)	346 (48.60)	257 (70.22)	109 (29.78)	366 (51.40)	375 (52.67)	337 (47.33)	712 (100)
Kooh-Sefid (ATSB treated barrier fences)	62 (63.27)	36 (36.73)	98 (45.79)	85 (73.28)	31 (26.72)	116 (54.21)	147 (68.69)	67 (31.31)	214 (100)
Percentage change	-47.46	-84.21	-71.68	-66.93	-73.28	-68.31	-60.80	-80.12	-69.94
Ratio change	1.90	6.34	3.53	3.02	3.52	3.16	2.55	5.03	3.33

Moreover, the *P*. *papatasi* population in the treatment villages with ATSB treated barrier fences in comparison with ASB treated barrier fences decreased several times (Tables 6 & 7).

**Table 7:** The comparison of *P*. *papatasi* frequency indoors and outdoors of Kooh-Sefid and Faraj-Abad using light traps (ATSB and ASB treated barrier fences), Markazi district, Qom province, 2015.

Site	Indoor			Outdoor			Total		
Site and type of Intervention	Male	Female	Total	Male	Female	Total	Male	Female	Total
	No. (%)	No. (%)	No. (%)	No. (%)	No. (%)	No. (%)	No. (%)	No. (%)	No. (%)
Faraj-Abad (ASB treated barrier fences)	88 (59.46)	60 (40.54)	148 (33.04)	198 (66.00)	102 (34.00)	300 (66.96)	286 (63.84)	162 (36.16)	448 (100)
Kooh-Sefid (ATSB treated barrier fences)	36 (52.94)	32 (47.06)	68 (54.84)	43 (76.79)	13 (23.21)	56 (45.16)	79 (63.71)	45 (36.29)	124 (100)
Percentage change	-59.09	-46.67	-54.05	-78.28	-87.25	-81.34	-72.38	-72.23	-72.32
Ratio change	2.45	1.87	2.18	4.60	7.85	5.35	3.62	3.60	3.61

however, this reduction was not statistically significant (p ≤ 0.116). Comparing the treatment village with the untreated one (Ali-Abad), based on the Kruskal Wallis Test, the mean rank of *P*. *papatasi* frequency in the treatment site reduced significantly. In addition, in pairwise comparison of the studied sites, a significant difference between the means of two groups of sand flies (ATSB and ASB treated barrier fences) was observed (p ≤ 0.024). In addition, although ATBS-treated barrier fences reduced sand fly densities more remarkably than ATSB sprayed on vegetation, the difference was not significant (p = 0.271).Also, the collected *P*. *papatasi* using light traps showed that the frequency of this species indoors and outdoors of Kooh-Sefid and Faraj-Abad (ATSB and ASB-treated barrier fences) had declined (Table 7). According to Tables 8 and 9, there was a decline in the Percentage and Ratio change of *P*. *papatasi* frequency indoors and outdoors in Jafar-Abad (plants, bushes, and shrubs sprayed with ATSB) and Kooh-Sefid (ATBS-treated barrier fences) compared to those of Ali-Abad (no intervention).

**Table 8:** The comparison of *P*. *papatasi* frequency indoors and outdoors in Jafar-Abad (plants, bushes, and shrubs sprayed with ATSB) and Kooh-Sefid (ATBS-treated barrier fences) with Ali-Abad (no intervention) using sticky traps, Markazi District, Qom province, 2015.

Site	Indoor			Outdoor			Total		
Site and type of Intervention	Male	Female	Total	Male	Female	Total	Male	Female	Total
	No. (%)	No. (%)	No. (%)	No. (%)	No. (%)	No. (%)	No. (%)	No. (%)	No. (%)
Ali-Abad (No intervention)	109 (29.70)	258 (70.30)	367 (32.31)	486 (63.20)	283 (36.80)	769 (67.69)	595 (52.38)	541 (47.62)	1136 (100)
Percentage change Jafar-Abad to Ali-Abad	-71.56	-65.50	-67.30	-68.72	-83.75	-74.25	-69.24	-75.05	-72
Percentage change Kooh-Sefid to Ali-Abad	-43.11	-86.05	-73.30	-82.51	-89.05	-84.91	-75.29	-87.62	-81.16
Raito change Jafar-Abad to Ali-Abad	3.52	2.90	3.06	3.20	6.15	3.88	3.25	4	3.57
Ratio change Kooh-Sefid to Ali-Abad	1.75	7.16	3.74	5.72	9.12	6.63	4.05	8.07	5.31

**Table 9:** The comparison *of P*. *papatasi* frequency indoors and outdoors in Jafar-Abad (plants, bushes, and shrubs sprayed with ATSB) and Kooh-Sefid (ATBS-treated barrier fences) with Ali-Abad (no intervention) using light traps, Markazi district, Qom province, 2015.

Site	Indoor			Outdoor			Total		
Site and type of Intervention	Male	Female	Total	Male	Female	Total	Male	Female	Total
	No. (%)	No. (%)	No. (%)	No. (%)	No. (%)	No. (%)	No. (%)	No. (%)	No. (%)
Ali-Abad (No intervention)	103 (37.05)	175 (62.95)	278 (34.49)	378 (71.59)	150 (28.41)	528 (65.51)	481 (59.68)	325 (40.32)	806 (100)
Percentage change Jafar-Abad to Ali-Abad	-66.02	-92.57	-82.73	-84.65	-71.33	-80.87	-80.67	-82.77	-81.51
Percentage change Kooh-Sefid to Ali-Abad	-65.05	-81.71	-75.54	-88.62	-91.33	-89.39	-83.57	86.15	-84.61
Raito change Jafar-Abad to Ali-Abad	2.94	13.46	5.79	6.52	3.49	5.23	5.17	5.80	5.41
Ratio change Kooh-Sefid to Ali-Abad	2.86	5.47	4.09	8.79	11.54	9.43	6.09	7.22	6.50

The incidence of the disease before and after intervention in treatment villages (Kooh-Sefid and Jafar-Abad) was 20.80% and 17.54% in 2014 and 4.81% and 6.44% in 2015, respectively. The comparison of ZCL incidence before and after the study showed that the ATSB method is effective as it decreased the disease incidence in treatment villages. Furthermore, this reduction was statistically significant (p = 0.011, 0.042). In 2015, 23 cases were infected. The mean age of the residents was 20.6 ± 13.7 and the most frequent age group was people above 15 (69.56%). Of the 23 patients under care, 43.47% were men and 56.53% were women. Most patients (65.21%) had one lesion, with hands and feet being the most common sites (78.26%). The highest disease prevalence was observed in the summer and fall due to the suitable climate conditions for sand flies and their seasonal activity peak. All specimens were identified by directly removing a smear from lesions and using the microscopic method. The results showed that the mortality rate from the bioassay test on ATBS-treated barrier fences for 5, 15, 30 and 45 days after spraying was 100, 95.83, 88.18 and 66.67%, respectively. The rate decreased to 50.83% after 60 days (Table 10).

**Table 10:** Results of the bioassay test on ATBS-treated barrier fences against *P*. *papatasi*, Markazi District, Qom province, 2015.

Day after application	Exposed sand flies with ATSB treated barrier fence	Dead sand flies exposed with ATSB			Mortality ± S.E. of sand flies exposed with ATSB
		Male	Female	Total	
5	120	47	73	120	100
15	120	39	76	115	95.83 ±4.35
30	120	32	64	106	88.18±6.24
Monthly mean	120	…	…	113.67	91
45	120	27	54	81	66.67±7.4
60	120	29	32	61	50.83±65
Monthly mean	120	…	…	71	58.75

Persistence and residual rate of ATBS-treated barrier fences effective substance in Qom Province climate (arid climate) was estimated at 45 days maximum. Thus, once every 45 days, barrier fences were impregnated with ATSB. In addition, barrier fences were surveyed every 15 days and, if needed, fixed.

## Discussion

This is the first report of ATSB application for *P*. *papatasi* control in Iran. The *P*. *papatasi* species is the main sand fly vector of ZCL in Iran [2]. In this research, bait solutions consisted of brown sugar, boric acid, and water. In the previous studies, brown sugar and boric acid, as attractive elements and oral toxin of the bait, had been used with ATSB [7,8]. The mechanisms through which ATSB destroy different insects include: toxic effects on their nervous system or abrading their insect exoskeletons (e.g. in cockroaches) [23]. Regarding the sand flies, previous studies have shown that their mid gut is poisoned and this, in turn, affects the metabolism [8]. Also it was previously proven that the boric acid toxin may also be transmitted to the sand fly via the tarsal response when in contact with ATSB [8]. So, this method targets the sugar seeking behavior of female and male sand flies. Plant sugars provide a considerable source of energy for females and are the only food source for males [24], and our study bears testimony to this fact. Both methods, ATBS-treated barrier fences and the ATSB applied on vegetation were effective in significantly decreasing the *P*. *papatasi* densities compared to the control. ATSB with the toxin boric acid successfully reduced the vector as an insecticide element of ATSB [6,7,8,25], ATBS-treated barrier fences and ATSB sprayed on vegetation effectively controlled *P*. *papatasi* in similar field sites in the arid habitats of the Jordan Valley and Israel [8]. Findings of the current study support erstwhile studies evaluating ATSB for sand fly control. In the previous studies [8], *P*. *papatasi* populations were reduced by 95% after ATSB application to natural habitats in Jordan Valley. Based on the results of other studies [7,8,25], ATSB application led to desired outcomes in controlling of most vectors, including malaria vectors, *Aedes*, and *Culex* in America, Africa and the Middle East. In Jafar-Abad (ATSB sprayed on vegetation), not only the Ratio change of *P*. *papatasi* population change reduced more than five times, this reduction was statistically significant. The Ratio change of *P*. *papatasi* population change reduced more than three times after the treatment in comparison with the previous year. Field studies [6,7,8,25] demonstrated of the effects of ATSB sprayed on vegetation in decline of the vectors. In our study area, dominant plant species was *T*. *aphylla*. In several studies [14], researchers have proven that flowering *Tamarix (Tamarix nilotica)* is a highly attractive sugar source for sand flies. Consequently, when sand flies feed on ATSB-sprayed *tamarix* flowers, they are easily caught and killed. In a similar study, *P*. *papatasi*, *P*. *sergenti* populations were reduced by 82.8% and 76.9% after spraying ATSB on non-flowering *tamarix* [26]. In this study, we showed that in Kooh-Sefid (ATBS-treated barrier fences), the Ratio change of *P*. *papatasi* population change reduced about eight times before and after treatment. The Ratio change of *P*. *papatasi* population reduced by more than three times compared to the control site, but this reduction was not statistically significant. Most probably, the ASB treated barrier fences themselves acted as a barrier to the movement of infected sand flies from rodent burrows toward human dwellings. It is hence predicted to be effective in decreasing the population of sand flies. This finding is concurrent with the results of a study on the abundance of sand flies by Müller et al (2011). As demonstrated in this study, when ATSB were applied on barrier fences, the oral toxin is ingested and therefore the vectors are exposed to the active ingredient for a long time and effectively killed. In addition, it is not necessary to have them landed on a bait for a long time or be sprayed directly. This study has also shown that the ATBS-treated barrier fences and ATSB sprayed on vegetation were not significantly different in terms of the results. Muller and et al observed that ATSB either sprayed on the vegetation or on barrier fences is an effective means against sand flies [8]. In addition, the comparison of the incidence of ZCL pre and post-study phases showed that the ATSB is an effective method in significantly decreasing the incidence of ZCL in treatment villages. With the decline in the abundance of *P*. *papatasi* in the study area, it is safe to expect the reduction of the number of infectious bites and the incidence of the disease. Based on the bioassay test, the residual rate of ATBS-treated barrier fences effective substance in Qom Province climate was estimated at 45 days at most. So, every 45 days, barrier fences were impregnated with ATSB. To the best of our knowledge, this method is cost-effective and efficacious for vector control.

## Conclusions

This vector control method might apparently work best in arid areas where attractive flowering plants are scarce. However, this study showed that ATSB can be successful in reducing sand fly populations in ZCL endemic areas. Further research is encouraged to evaluate the effects of other insecticides in ATSB for controlling of *p*. *papatasi* in endemic areas of Iran.

## Supporting information

S1 Data Raw data used for this study. (XLSX)
